# A New Sight of Ozone Usage in Textile: Improving Flame Retardant Properties

**DOI:** 10.3390/polym16060735

**Published:** 2024-03-07

**Authors:** Semiha Eren, İdil Yiğit, Kadriye Kutlay, Zehra Kaya, Cansu Basrık, Hüseyin Aksel Eren

**Affiliations:** 1 Textile Engineering Department, Bursa Uludag University, Bursa 16059, Türkiye; semihaeren@uludag.edu.tr (S.E.); aksel@uludag.edu.tr (H.A.E.); 2 Textile, Clothing, Footwear and Leather Department, Bursa Uludag University, Bursa 16980, Türkiye; 3 Berteks Company, Bursa 16245, Türkiye; kadriye.kutlay@berteks.com (K.K.); zehra.kaya@berteks.com (Z.K.); arm11@berteks.com (C.B.)

**Keywords:** ozone, flame retardant (FR), IMO, flammability, polyester

## Abstract

Ozone, widely recognized as an environmentally friendly gas, is extensively used in various textile industry applications. These include pre-treatment processes like bleaching and desizing, as well as creating pattern and vintage effects, wastewater clarification, and surface modification. This study focuses on ozone as a novel solution to a specific challenge: addressing the reduction in flame retardancy properties experienced by flame-retardant (FR) polyester fabrics during post-treatment processes in the production line. Experimentation involved subjecting the fabrics to ozonation and exploring different combinations of ozone flow rates and treatment durations. Mechanical and functional properties of the fabrics were examined, with flammability tested according to International Maritime Organization (IMO) standards. Notably, treatment with a 5 L/min ozone flow rate, a 7.01 g/h ozone concentration ratio, and a duration of 10 min showed significant improvements in IMO values, ensuring compliance with required standards. Furthermore, treated samples underwent comprehensive tests for fastness and strength, yielding results within acceptable ranges. Fourier-transform infrared (FT-IR) and thermogravimetric analysis (TGA) measurements were conducted to evaluate the impact of ozonation. FT-IR results indicated that the presence of C-H groups associated with dyestuff contributed to decreased flame retardancy in the original fabric post-dyeing. However, these groups were effectively eliminated through ozonation, thereby enhancing the fabric’s flame retardancy.

## 1. Introduction

Flammability presents a significant risk to both human lives and property, making it a pressing concern in our daily lives [1,2]. The burning behavior of textile products, widely used across various fields, is characterized by their susceptibility to ignition and sustained burning [3]. These materials, due to their hydrocarbon chains, tend to emit heat, flames, and smoke when exposed to fire [1,4]. To address this issue, flame retardants are incorporated into polymeric materials to prevent fire onset or restrict its propagation [5]. Since the 1950s, flame retardant chemicals have been developed for fibers and textiles [6]. However, many of these chemicals have shown toxic effects on humans and the environment. Consequently, researchers are actively exploring novel flame retardant finishes and coatings with improved environmental profiles and reduced toxicity for textile applications [7]. There is a growing interest in developing flame retardants derived from renewable and sustainable sources, reflecting a broader shift toward more sustainable and eco-friendly solutions in flame retardancy [8]. Various methods, including ASTM standards, have been developed to assess fabric flammability [9,10]. For materials lacking inherent flame retardancy, such as meta/para-aramid, glass fiber, carbon fiber, and fluorocarbon fiber, different approaches are employed to impart flame retardant properties:

(i) Fiber structures can be modified through copolymerization and chemical modification techniques involving the addition of halogen and/or phosphorus-containing comonomers during copolymerization.
(ii) Flame retardant chemicals can be added to the synthetic polymer during the fiber spinning process.
(iii) Flame retardancy can be achieved through finishing treatments, wherein fabrics are treated with flame-retardant chemicals [11,12,13,14].
(iv) Nanoparticles with flame-retardant properties can be incorporated into coatings or applied directly to fibers to enhance their resistance to ignition [15].
(v) Phase change materials (PCM) can be integrated into fabrics to provide additional flame resistance while maintaining wearer comfort [16,17].

Polyester, a widely used synthetic polymeric material in textiles, poses significant risks due to its highly flammable nature and tendency to drip. Therefore, flame-retardant properties are essential for polyester products to meet customer demands and comply with government regulations [9,10,18,19,20].

Apart from factors like fiber content, fabric construction, and environmental conditions, the choice of finishing materials can impact fabric flammability [21]. The hydrophobic nature of polyester fibers results in relatively low chemical absorption during wet process treatments, potentially decreasing flame retardancy [22]. For instance, softeners in fabrics have been found to reduce flame retardancy [2,23].

Even when raw materials exhibit satisfactory flame retardancy, finishing processes can lead to strength loss or increased flammability due to flammable chemicals present on the fabric’s surface [21]. Researchers have highlighted the adverse effects of dyes, particularly disperse azo dyes, on polyester fabric flammability, as these dyes tend to migrate to the fiber’s surface during heat treatment [23,24].

Disperse dyes, with their limited solubility in water, can aggregate and deposit on the surface of PET fibers during the dyeing process. If not effectively removed, this surface contamination can negatively impact various fabric properties, including flame retardancy [23,24,25,26,27]. The conventional method for clearing these dyes involves reduction clearing, breaking down residual disperse dye molecules into smaller, colorless, and more readily water-soluble fragments [26,27].

Oxidative agents like ozone (O_3_) have shown effectiveness in breaking down deposited disperse dyes on fiber surfaces. Ozone, industrially produced using commercially available ozone generators, has a high oxidation potential and has successfully decolorized disperse dyeing effluents through ozone treatment [28,29,30]. Ozone gas is increasingly favored for various industrial applications due to its potent oxidative properties, offering a more environmentally friendly and energy-efficient alternative to traditional processes in the textile industry [28,29,30,31].

As demonstrated by previous studies, the flame retardancy of fabrics can diminish following dyeing, washing, and fixation. Subsequent finishing processes are often required to enhance the flame retardant properties. However, reusing chemicals in these processes increases costs and impacts the environment [32]. Therefore, it is advantageous to counteract the decrease in flame retardancy through ozone treatment without harming the environment or necessitating the use of additional chemicals. Thus, the primary objective of this study is to restore the diminished flame retardancy resulting from these processes through ozone gas treatment. This innovative approach holds significant promise for the textile industry, offering a more sustainable and environmentally friendly method and effectively clearing the surface of polyester fabrics.

## 2. Materials and Methods

In this study, 100% polyester fabrics made of polyethylene terephthalate (PET) were used. The fabrics were produced using flame retardant polyester yarn containing comonomer with phosphorus. The polyester yarns (300/288 centered and 75/72 550 Draw Texture Yarn) were sourced from Toray/Korea company. To achieve the desired coloration, the yarns were dyed using a mixed dyestuff comprising Color Index (CI) Disperse Orange 30 (0.02%) (by DyStar, Tekirdağ, Türkiye), CI Disperse Red 167.1 (0.002%) (by DyStar), and Terasil Blue LF (0.02%) (by Hunstman, The Woodlands, TX, USA) in a Thies dyeing machine. The dyeing recipe included 1.5% dispersant (Levanol HDL by Genkim, Bursa, Türkiye), 0.4% leveling agent (Segacar D Liq by Sözal Kimya, Bursa, Türkiye), 0.3 g/L wetting agent (Revapol ENN by Alfa Kimya, Bursa, Türkiye), and 0.05 g/L acid buffer (Exapon Cn Plus by MaiTürk Kimya, Bursa, Türkiye).

After dyeing, the yarns were neutralized with acid (Exapon CN Plus). Since the fabric required a light color, no reduction-clearing process was performed. The yarns were woven as jacquard weaves with double in the warp direction satin 5 (Satin 4/1) using a Stäubli, Horgen, Switzerland weaving machine.

The resulting fabric samples underwent processing through a Monforts Montex 5000 model Stenter, Mönchengladbach, Germany (20 m/min) and a Mathis Brand Laboratory type mini Stenter, Oberhasli, Switzerland (LABDRYER TYPE “LTE”) (1 min) according to the designated method.

For ozonation, a ProOzon (Ankara, Türkiye) generator with a capacity of 25 g/h was utilized. The ozonation process occurred in an aqueous environment, utilizing an ATAC BB01F (Atac Co., Istanbul, Türkiye) model bobbin dyeing machine modified to accommodate the gas. The outlet gas of the ozone generator was integrated into the liquor circulation line of a sample Atac BB01F sample dyeing machine via a venturi injector. The system’s setup can be referenced in the works of Yigit and Eren 2017 and Eren and Yetişir 2018 [33,34].

The methods employed in this study are divided into two main parts. The first part covers fabric production and its associated treatments, while the second part is dedicated to test methods.

### 2.1. Production and Treatments

#### 2.1.1. Yarn Dyeing

**Figure 1:**
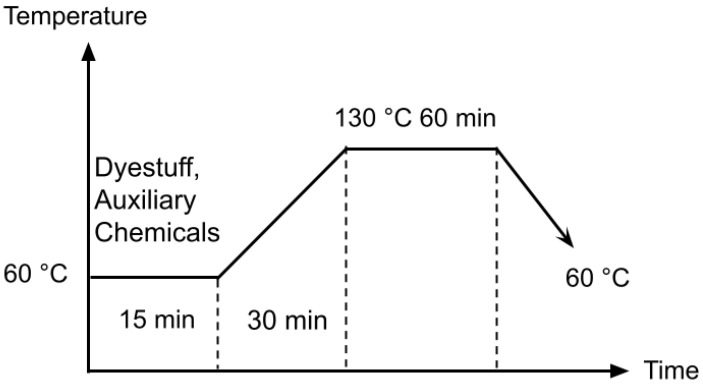
Dyeing diagram of flame retardant polyester yarns.

The flame-retardant polyester yarns underwent the standard polyester dyeing process and were subsequently transferred to the SSM PW1 model winding machine (Wädenswil, Switzerland). Figure 1 illustrates the dyeing diagram for flame-retardant polyester yarns.

#### 2.1.2. Fabric Production

The yarns were woven into a jacquard weave, resulting in 36 weft/cm and 80 warp/cm density fabric weighing 660 g/L meters.

#### 2.1.3. Ozonation

**Figure 2:**
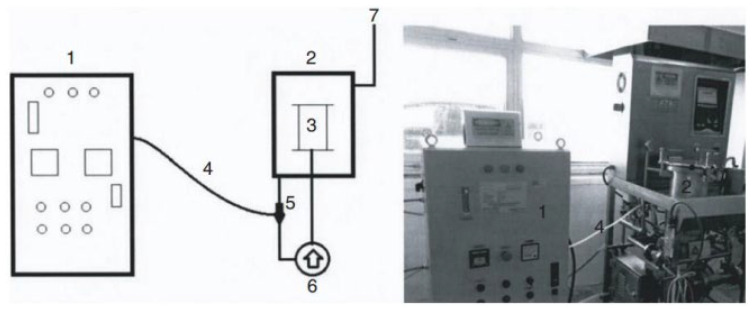
Ozone generator and integrated sample dyeing machine (1. ozone generator; 2. autoclave; 3. beam; 4. ozone flow pipe; 5. venturi injector; 6. circulation pump; 7. ozone outlet) [26].

The ozonation process was conducted in an ATAC BB01F sample bobbin dyeing machine specialized and modified for ozonation (refer to Figure 2) at room temperature conditions (22 ± 2 °C) using water, without the addition of any chemicals. Water (liquor ratio of 15:1) was maintained, and optimization experiments were carried out to determine the appropriate ozonation time and ozone flow. After the treatments, the fabrics were fixed at 150 °C.

The ozone concentration was measured using the standard iodometric method (APHA Standard Methods 2350 E) [35], yielding a 7.01 g/h value at a 5 L/min ozone gas flow rate.

#### 2.1.4. Washing

The raw fabric underwent two wash cycles in a BABCOCK washing machine, using 10 g/L caustic (by Ozan Kimya, Bursa, Türkiye), 15 g/L reductant (LAUCOL RX by Erca, Grassobbio, Italy), and 10 g/L oil remover (OPTISOL RG-50 by Optima Kimya, Tekirdağ, Türkiye), with continuous washing at 80 °C.

#### 2.1.5. Fixation

The fabric was subsequently dried in a Monforts Montex 5000 machine (Mönchengladbach, Germany) at 150 °C at a 15 m/min speed. 

### 2.2. Test Methods

#### 2.2.1. Flame Retardant Test

The majority of standardized testing methods employed to assess flammability and flame resistance are tailored for measuring specific flammability characteristics of textile materials [36]. The International Maritime Organization (IMO) has established the Fire Test Procedure Code Part 7 (IMO FTPC P7) [37] as a standard for testing flame retardancy developed by the organization itself. This code outlines the methods for evaluating the flame retardant properties of various ship components, with Part 7 specifically addressing the testing of flame retardancy on vertical textile surfaces or films.

In essence, the method involves suspending samples vertically for testing, with dimensions of 220 mm × 170 mm. A key criterion is assessing whether the drips produced during the test can ignite other materials on the surface. To evaluate this, a piece of cotton, dried at 100 °C to eliminate moisture, is positioned 15 cm below the sample. A 40 mm propane gas flame is then used for ignition, with cotton serving as a reference material due to its well-known flammability characteristics.

**Figure 3:**
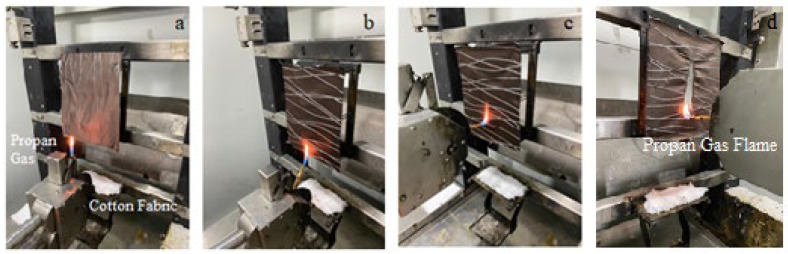
IMO test method: (**a**) test device, (**b**) edge (45°) flame, (**c**) surface (90°) flame, (**d**) hole caused by burning or flaming.

To initiate the testing process, four samples are prepared in both width and length directions. For each direction, two flame orientations, referred to as edge and surface in the standard, must be applied. In the edge direction, the flame makes contact with the lower edge of the fabric at a 45-degree angle, while in the surface direction, it contacts the front surface of the fabric at a 90-degree angle. Figure 3 provides a visual representation of the IMO test method.

For fabric directions and flame orientations, two different flame contact times are tested: 5 and 15 s. Therefore, fabric samples in both directions are assessed in four distinct ways.

The following observations are recorded during the testing:

Ignition Time: After the flame application period concludes, the flame source is removed. If there is any ignition that persists after the flame source is removed, its duration is documented, and this duration should not exceed 5 s.

Dripping: It is crucial to note if any ignition occurs in the cotton placed beneath the sample. No ignition of the cotton should result from dripping from the fabric.

Hole Size: The length or width of the hole caused by burning or flaming in the sample after the test must not exceed 150 mm. This requirement is consistently met in all tests covered by this article and is therefore omitted from the tables [38,39].

After conducting tests on fabric samples, the two samples displaying the poorest results in both the weft and warp directions were selected. Additionally, five more samples were chosen for testing in each direction, maintaining the same conditions as the initial samples. The testing was carried out using the James Heal Flexiburn test device in the Berteks Textile Physics Laboratory.

This study calculated the average value of the test result times. The primary objective was not to achieve exceptional test results but rather to showcase an improvement in flame retardancy levels after ozonation. In the IMO Part 7 test standard, the average value for after-flame time is typically not considered; any sample flaming for more than 5 s is considered failure. However, for this study, a modification was made to the evaluation process while applying the test method without any alterations. This adjustment aimed to clearly illustrate the difference between the trials. Therefore, the average value of the after-flame time was taken into account, providing a more transparent demonstration of the improvement between the samples that failed the test.

#### 2.2.2. Fourier-Transform Infrared Spectroscopy (FT-IR)

FT-IR analysis provides information about the functional groups in molecules and the vibrational frequencies of various bonds. FT-IR analysis was performed on the samples to determine the reason for the increased flame retardancy test success after ozonation. The FT-IR structure analysis was conducted using the SHIMADZU Tracer IR100 (Japan) brand spectrophotometer test device.

#### 2.2.3. Thermogravimetric Analysis (TGA)

A thermogravimetric analysis (TGA) test was conducted on the SDTQ600 TA with ISO 11358-1 [40]. The temperature range of the TGA was 20 °C to 600 °C with a heating rate of 20 °C/min under a nitrogen atmosphere and then heated from 600 °C to 900 °C under an oxygen atmosphere.

#### 2.2.4. Measurement of Color

Color measurements were conducted to evaluate the potential impact of ozonation on the color of the samples, alongside the improvement in flame retardancy test success. The color difference tests were carried out using a Datacolor brand spectrophotometer device and the Kavolab ColorOffice program. A comprehensive set of eight measurements was taken on both the front and back sides of the samples to ensure a thorough assessment of any color variations resulting from the ozonation process.

#### 2.2.5. Fastness Tests

Fastness tests were conducted to examine the effect of ozonation on the fastness values of the samples. The washing fastness was evaluated using a thermal oil paint machine, following the ISO 105 C06 standard [41]. The rubbing fastness tests were performed using the J. Heal Crockmeter, by the ISO 105 X12 standard [42].

#### 2.2.6. Strength Tests

Tensile strength tests were carried out to evaluate the effects of ozonation on the strength values of the samples. The fabric samples were subjected to strength tests using the Titan 3 device, following the EN ISO 13934-1 standard [43]. A pre-stress of 2 N was applied, followed by a load of 100 N.

### 2.3. Optimization

This collaborative study was conducted in partnership with Berteks Textile Company (Bursa, Türkiye) to address the challenge of low flame retardancy test success in a fabric highly favored by their customers. Despite being woven with flame-retardant yarn, the fabric experienced a decrease in test success due to the presence of impurities such as oil and dye. These impurities, inadequately removed during yarn dyeing, yarn transfer, and weaving stages, resulted in incomplete elimination, reducing flame retardancy. Among 50 different lots dyed at various times, 20 failed the flame retardancy test, underscoring the issue of incomplete impurity removal from the fabric.

In pursuit of optimization, various ozonation times and ozone flow rates were tested on fabrics acquired from different production stages. Flammability tests (IMO) were conducted on the resultant samples. The trials revealed that the most favorable results were achieved with an ozonation time of 10 min and a flow rate of 5 L/min.

The study categorized the investigation into four distinct categories, aiming to identify which process contributes to the reduction of the flame-retardant effect in fabrics. The flame retardancy of these fabrics was then assessed using the IMO test, with the results detailed in Table 1. These categories are:

Category 1: Undyed fabrics with flame-retardant additives (FR). It aimed to assess the effectiveness of the FR additives in the yarns.

Category 2: Fabrics produced from dyed (D) yarns with flame retardant (FR) additives (FR+D). The objective was to observe the effects of dyeing processes.

Category 3: Fabrics produced from dyed (D) yarns with flame retardant (FR) additives that were subsequently washed (W) and fixed (F) (FR + D + W + F). This category aimed to see the flame retardant effect after all processes.

Category 4: The process in which ozonation (O) is performed after the processes carried out in Category 3. This category of fabrics was produced from dyed (D) yarns with flame retardant (FR) additives that were subsequently washed (W), fixed (F), and ozonated (O) (FR + D + W + F + O).

## 3. Results

The results of the optimization process involving ozone gas and the corresponding combustion data are presented in Table 1. Various ozone flow rates and durations were tested, employing a flow rate of 5 L/min and an ozonation time of 10 min. The flame retardancy effect, which had reduced after dyeing, washing, and fixing fabric produced with flame retardant (FR) additives, demonstrated successful improvement after ozonation.

To evaluate the fabrics, the average flame time and the number of burning drips were measured in a total of 18 tests, considering both surface and edge directions in both weft and warp orientations. Initially manufactured with dyed yarns and FR additives, the fabrics underwent a standard washing and fixing procedure upon arrival at the facility. These optimized conditions aim to enhance flame retardancy while preserving the desired physical properties of the fabrics.

Practical experience and the literature affirm that the flame retardancy properties of fabrics often decrease after dyeing, washing, and fixing processes. However, it is imperative to ensure that while the flame retardancy test results improve, the physical performance tests of the samples also meet acceptable standards. A comprehensive set of tests, including color measurement, fastness, and strength tests, were conducted to address this.

Additionally, FTIR measurements were performed to elucidate the factors contributing to the increase in flame retardancy test success. Category 3 (FR added-dyed Fabric by Washed and Fixed Unprocessed) is the reference sample used for comparing the results. This reference sample serves as a baseline for evaluating the impact of different processes and treatments on flame retardancy and physical performance.

### 3.1. Flame Retardant Test Results

Upon examining Table 1, the IMO test results for Category 1 were successful as expected, given that no processes other than the addition of flame retardant (FR) were applied to the samples. However, in Category 2 and Category 3, subsequent processes such as dyeing, washing, and fixing led to a loss of flame retardant properties in the fabrics. The literature supports the idea that washing, mainly due to the presence of softeners and lubricants on the surface, diminishes flammability. Additionally, as supported by the data obtained, the thermomigration of dyes at high fixing temperatures contributes to this loss. Hence, it is affirmed that fabrics with FR additives should undergo washing and fixing steps, as seen in Category 3.

In Category 4, where ozonation was implemented, the fabrics regained flame retardancy, with 5 s or less ignition times. Notably, Category 2 and Category 3 exhibited cotton ignition and flaming in the edge direction during combustion tests, while the Category 4 fabric showed no drips, and the average flaming time was 3 s. This data supports the conclusion that the ozonation process can be applied after washing and fixing procedures for the fabrics.

FTIR measurements were conducted to elucidate the increase in flame retardancy test success. The results further supported the efficacy of the ozonation process.

### 3.2. FT-IR Test Results

**Figure 4:**
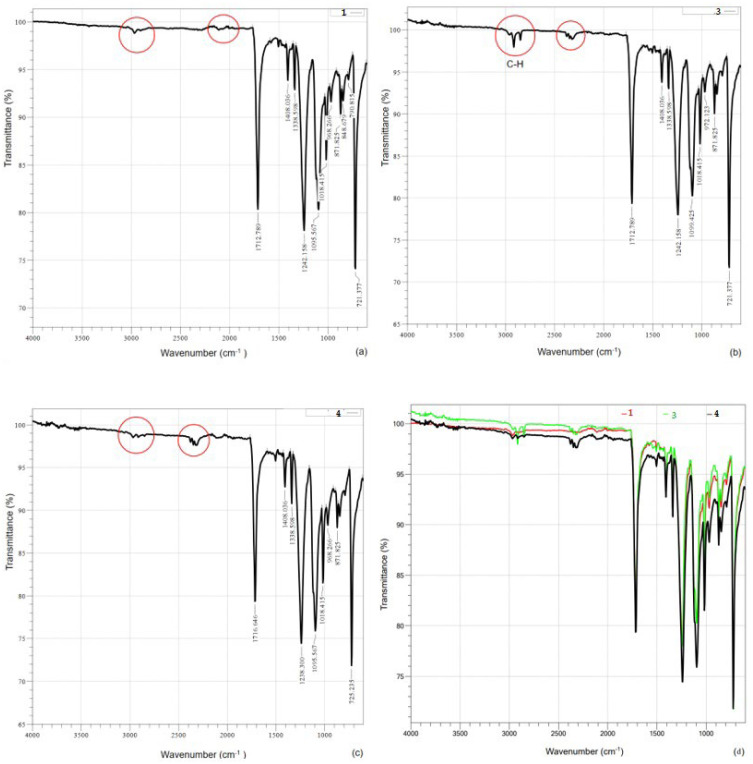
FTIR spectra analyses of samples: (**a**) Category 1: FR, (**b**) Category 3: FR + D + W + F, (**c**) Category 4: FR + D + W + F + O, (**d**) Comparative graph of FTIR samples (C1, Category 1—red line; C3, Category 3—green line; C4, Category 4—black line). FR, Undyed FR-added Fabric); FR + D + W + F (FR added-Dyed Fabric by Washed and Stenter Fixed Unprocessed); FR + D + W + F + O (FR added-Dyed Fabric by Washed, Stenter Fixed and Ozonated). Red circles show C-H groups that present by dyeing and affected by ozone treatment.

Figure 4 illustrates the mechanism of dye and ozonation with the FT-IR spectra analysis. Upon examination of the results, it was noted that the peak of the C-H bond at 3000–2840 cm^−1^ is absent in the flame retardant undyed (Category 1) sample, whereas it is present in the flame retardant dyed (Category 3–FR added-Dyed Fabric by Washed and Fixed Unprocessed) sample. These C-H bonds are attributed to the dyestuff groups used in dyeing. Following the ozonation process, it was observed that the same peak disappeared again, similar to the Category 1 sample. This indicates that the ozonation process effectively removes the dyestuff from the surface. The disappearance of these peaks, along with the absence of the C-H peak after the ozonation process, provides supporting evidence for the effectiveness of ozonation. Ozone plays a pivotal role in the degradation of disperse dyestuffs [44]. The improvement in test success post-ozonation is further substantiated by the elimination of dyestuff groups that may contribute to combustion.

### 3.3. Thermogravimetric Analysis Results

**Figure 5:**
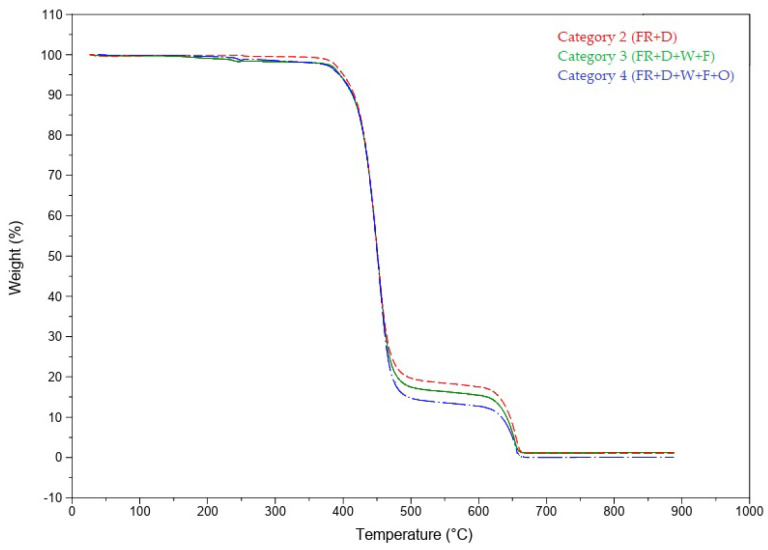
TGA curve of samples.

Thermogravimetric analysis (TGA) is a technique used to study the thermal degradation behavior of polymers by measuring the weight loss of a material against an increase in temperature. TGA provides crucial information on thermal properties such as the onset of decomposition, decomposition temperature, and decomposition rate. Figure 5 illustrates the TGA curves of the fibers, categorized into three groups. The weight percentage (wt %) of the retained mass as the temperature increases is depicted in the graphs. Remarkably, the ozone-treated samples (Category 4) showed a slightly higher initial degradation temperature of the polymer (413.16 °C) compared to the untreated samples in Category 2 (410.24 °C) and Category 3 (411.58 °C). However, no significant differences among the categories were observed in the residual samples.

Upon examining the TGA results, no substantial differences were noted among the samples. This can be attributed to the absence of any additional chemicals added to the structure or surface of the material for improved flame retardancy. Therefore, the ozone treatment performed under the selected parameters did not significantly change the polymer’s structure. Ozonation primarily affects the material’s surface without altering its internal structures. Consequently, as no operations were conducted to modify the internal structures of the samples, it is expected that the TGA results do not show significant variations.

### 3.4. Physical Test Results

As the flame retardancy test’s success improves, it is crucial to ensure that the physical performance tests of the samples also fall within acceptable limits. A comprehensive set of tests, including color measurement, fastness, and strength tests, were conducted on the samples to achieve this objective.

#### 3.4.1. Color Measurement Results

**Figure 6:**
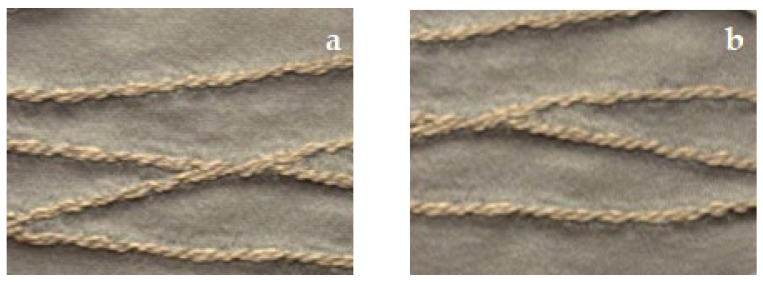
Image of samples before and after ozone treatment. (**a**) Category 3 (before), (**b**) Category 4 (after).

Upon analyzing the color measurement results of the samples, as outlined in Table 2, it was observed that the ozonation process did not induce a notable change in the color values. The color measurement results met the success criterion of ΔE ≤ 1. No significant differences were noted in the values related to lightness–darkness (ΔL), green–redness (Δa), and yellowness–blueness (Δb) of the samples following the treatment. Images in Figure 6 supported the color measurement results of samples.

#### 3.4.2. Fastness Test Results

When examining the washing and rubbing fastness values as presented in Table 3, it became evident that the fastness values of the Category 4 samples remained consistent with the reference samples, indicating that the ozonation process did not have an impact on the fastness values.

After undergoing 5 wash cycles, the samples were observed to maintain their flame-retardant effect.

#### 3.4.3. Strength Test Results

The strength values of the samples are presented in Table 4. The tensile strength values of Category 4 exhibited 3% decrease compared to the reference sample (Category 3). These results fall within the acceptable tolerance values set by companies.

## 4. Conclusions

The flame-retardant polyester fabrics, manufactured using flame-retardant yarns, often experience a reduction in flame retardancy after undergoing dyeing, washing, and fixation processes. Previous studies attribute this decline to the influence of dyestuffs, softeners, and lubricants. In this study, samples with diminished flame retardancy underwent ozonation treatment. Following a 10 min ozonation process at a rate of 5 L/min, flame retardant tests (IMO) were conducted again. Significantly, Category 4 samples (FR-added Dyed Fabric by Washed, Fixed, and Ozonated) showed remarkable improvements in both IMO and physical tests. The breakdown of dyestuffs by ozone, whether present on the surface or remaining post-processing, facilitated the enhancement of flame-retardant properties. FT-IR analyses revealed the disappearance of dye groups associated with ignition after ozonation. Samples yielding optimal test results also demonstrated performance in terms of minimal color differences (ΔE < 1), excellent fastness (5 degrees), and acceptable strength (3% decrease). The application of ozone gas, known for its environmentally friendly nature, effectively enhanced the success of flammability tests without necessitating additional flame retardant finishing on FR-added yarn samples. This method proved effective even for samples initially experiencing decreased test success after undergoing other processes. The primary aim of this study is to introduce ozone treatment as an innovative approach to address the documented loss of flame retardancy in the literature. By utilizing environmentally friendly methods like ozone, manufacturers can mitigate environmental and health risks associated with using additional chemicals, thereby contributing to creating more sustainable products.

## Figures and Tables

**Table 1:** IMO test results after different process steps.

Sample	Ignition Time (s)	Dripping Number	Observation *	Result
Category 1 (FR)	-	-	-	Accept
Category 2 (FR + D)	8.3	5	EDGE/5 Times	Reject
Category 3 (FR + D + W + F)	14	6	EDGE/6 Times	Reject
Category 4 (FR + D + W + F + O)	3	0	-	Accept

The table displays data obtained from a 10 min ozonation process at a 5 L/min ozone gas flow. The codes used correspond to the initial letters of the processes as follows: FR: Flame Retardant; FR + D: Flame Retardant + Dyed; FR + D + W + F: Flame Retardant, Dyed, Washed, Fixed; FR + D + W + F + O: Flame Retardant, Dyed, Washed, Fixed, and Ozonated. The data are presented in the format: * (Ignition Direction And Cotton Ignition Times).

**Table 2:** Color measurement results of Category 4 (FR added-Dyed Fabric by Washed, Stenter Fixed and Ozonated) samples.

Sample Name	ΔL	Δa	Δb	ΔE
Category 4 (FR + D + W + F + O)	0.38	0.50	0.38	0.85

The Reference Sample is Category 3 (FR added-Dyed Fabric by Washed and Stenter Fixed Unprocessed).

**Table 3:** Washing and rubbing fastness results of Category 4 samples.

Sample Name	Rubbing Fastness		Washing Fastness					
	Dry	Wet	Acetate	Cotton	Polyamide	Polyester	Acrylic	Wool
Category 3 (FR + D + W + F)	5	5	5	5	5	5	5	5
Category 4 (FR + D + W + F + O)	5	5	5	5	5	5	5	5

The Reference Sample is Category 3 (FR added-Dyed Fabric by Washed and Stenter Fixed Unprocessed). Category 4 (FR added-Dyed Fabric by Washed, Stenter Fixed and Ozonated).

**Table 4:** Tensile strength results of Category 4 samples.

Sample Name	Tensile Strength	
	Force (N)	Elongation (%)
Category 3 (FR + D + W + F)	1177.33	41.47
Category 4 (FR + D + W + F + O)	1140.84	38.83

The Reference Sample is Category 3 (FR added-Dyed Fabric by Washed and Stenter Fixed Unprocessed).

## Data Availability

All data relevant to the study are included in the article. On a reasonable request, additional data shall be available from the corresponding author.
